# SYNTAXIN OF PLANTS 132 underpins secretion of cargoes associated with salicylic acid signaling and pathogen defense

**DOI:** 10.1093/plphys/kiae541

**Published:** 2024-10-10

**Authors:** Sakharam Waghmare, Lingfeng Xia, Thu Phan Ly, Jing Xu, Sahar Farami, Richard Burchmore, Michael R Blatt, Rucha Karnik

**Affiliations:** Plant Science Group, School of Molecular Biosciences, College of Medical, Veterinary and Life Sciences, University of Glasgow, Bower Building, University Avenue, Glasgow G12 8QQ, UK; Plant Science Group, School of Molecular Biosciences, College of Medical, Veterinary and Life Sciences, University of Glasgow, Bower Building, University Avenue, Glasgow G12 8QQ, UK; Plant Science Group, School of Molecular Biosciences, College of Medical, Veterinary and Life Sciences, University of Glasgow, Bower Building, University Avenue, Glasgow G12 8QQ, UK; Plant Science Group, School of Molecular Biosciences, College of Medical, Veterinary and Life Sciences, University of Glasgow, Bower Building, University Avenue, Glasgow G12 8QQ, UK; Plant Science Group, School of Molecular Biosciences, College of Medical, Veterinary and Life Sciences, University of Glasgow, Bower Building, University Avenue, Glasgow G12 8QQ, UK; School of Infection & Immunity, College of Medical, Veterinary and Life Sciences, University of Glasgow, Sir Graeme Davies Building, Gilmorehill Campus, University Place, Glasgow G12 8QQ, UK; Laboratory of Plant Physiology and Biophysics, University of Glasgow, Bower Building, Glasgow G12 8QQ, UK; Plant Science Group, School of Molecular Biosciences, College of Medical, Veterinary and Life Sciences, University of Glasgow, Bower Building, University Avenue, Glasgow G12 8QQ, UK

## Abstract

Secretory trafficking in plant cells is facilitated by SNARE (soluble *N*-ethylmaleimide-sensitive factor attachment protein receptor) proteins that drive membrane fusion of cargo-containing vesicles. In Arabidopsis, SYNTAXIN OF PLANTS 132 (SYP132) is an evolutionarily ancient SNARE that functions with syntaxins SYP121 and SYP122 at the plasma membrane. Whereas SYP121 and SYP122 mediate overlapping secretory pathways, albeit with differences in their importance in plant–environment interactions, the SNARE SYP132 is absolutely essential for plant development and survival. SYP132 promotes endocytic traffic of the plasma membrane H^+^-ATPase AHA1 and aquaporin PIP2;1, and it coordinates plant growth and bacterial pathogen immunity through PATHOGENESIS-RELATED1 (PR1) secretion. Yet, little else is known about SYP132 cargoes. Here, we used advanced quantitative tandem mass tagging (TMT)-MS combined with immunoblot assays to track native secreted cargo proteins in the leaf apoplast. We found that SYP132 supports a basal level of secretion in Arabidopsis leaves, and its overexpression influences salicylic acid and jasmonic acid defense-related cargoes including PR1, PR2, and PR5 proteins. Impairing SYP132 function also suppressed defense-related secretory traffic when challenged with the bacterial pathogen *Pseudomonas syringae*. Thus, we conclude that, in addition to its role in hormone-related H^+^-ATPase cycling, SYP132 influences basal plant immunity.

## Teaser

SYNTAXIN OF PLANTS 132-mediated traffic dictates basal secretory proteome profiles in Arabidopsis and influences plant immunity.

## Introduction

Secretory traffic, or the extracellular release of cargoes, including signaling molecules, antimicrobials, and cell wall materials to the apoplast underpins plant cell expansion for growth and cellular homeostasis, and it impacts pathogen defense. Secretory vesicle traffic is primarily mediated by the so-called SNAREs (soluble *N*-ethylmaleimide-sensitive factor attachment protein receptors) of plants that drive the fusion of secretory cargo-containing vesicles at the plasma membrane (PM) through binding of t-SNAREs localized to the target membrane with vesicle-localized v-SNAREs in a complex (Bassham et al. 2008; Kwon et al. 2008, 2020). The minimal SNARE core complex constitutes a highly conserved glutamate (Q) motif, contributed by the Qa-, Qb-, Qc-SNAREs, and the arginine (R) motif, on R-SNAREs (Fasshauer et al. 1998; Bock et al. 2001; Bassham and Blatt 2008).

In Arabidopsis (*Arabidopsis thaliana*), Qa-SNARE SYNTAXIN OF PLANTS 121 (SYP121), also known as SYNTAXIN RELATED PROTEIN 1 (SYR1) or PENETRATION1 (PEN1) (Leyman et al. 1999), and its homolog SYNTAXIN OF PLANTS 122 (SYP122) are both expressed constitutively throughout the vegetative plant (Enami et al. 2009). They bind within SNARE core complexes with the Qbc-SNARE SYNAPTOSOMAL-ASSOCIATED PROTEIN 33 kDa (SNAP33) and R-SNARE VESICLE-ASSOCIATED MEMBRANE PROTEINs VAMP721 and VAMP722 and mediate secretory traffic at the PM (Kwon et al. 2008; Karnik et al. 2013; Zhang et al. 2015, 2017; Waghmare et al. 2018). SYP121 and SYP122 share common secretory functions, but they are also each involved in the trafficking of distinct cargo subsets (Waghmare et al. 2018). A substantial body of work with SYP121 trafficking cargoes establishes the Qa-SNARE as essential for growth, programmed stomatal closure, and abiotic stress tolerance (Leyman et al. 1999; Eisenach et al. 2012; Karnik et al. 2015, 2017; Waghmare et al. 2018; Larson et al. 2020).

Qa-SNARE SYNTAXIN OF PLANTS 132 (SYP132) is an evolutionarily ancient SNARE that is expressed throughout the plant during all developmental stages. It accumulates as a newly synthesized protein in the cell division plane and drives vesicle traffic at the phragmoplast for cytokinesis (Sanderfoot et al. 2000; Enami et al. 2009; Park et al. 2018). SYP132 is thought to be important for basal secretion in the vegetative plant (Sanderfoot et al. 2000; Collins et al. 2003; Bassham and Blatt 2008; Ichikawa et al. 2014). SYP132 exhibits diverse biological roles in plant biotic interactions including bacterial pathogenesis in various plant species such as *A. thaliana*, *Nicotiana tabacum*, and *Triticum aestivum* (Kalde et al. 2007; Wang et al. 2014; Baena et al. 2022) and arbuscular mycorrhizal symbiosis in *Medicago truncatula* (Huisman et al. 2020). The SYP132 SNARE core-complex assembly for vesicle fusion is regulated by SEC1B (Karnahl et al. 2018), a putative paralog of Sec1/Munc18 protein SEC11 that preferentially regulates the SNARE SYP121 (Karnik et al. 2013, 2015). Unusually, SYP132 also promotes endocytic traffic, influencing abundance and activities of PM proton (H^+^)-ATPase1 (AHA1) and aquaporin PIP2;1 at the cell membrane (Xia et al. 2019, 2020; Baena et al. 2022, 2024).

Studies in *Nicotiana benthamiana* first showed that suppressing *NbSYP132* expression suppresses the immunity against bacterial pathogens, attributed largely to the reduced extracellular accumulation of antimicrobial PATHOGENESIS-RELATED 1 (PR1) protein in the apoplast (Kalde et al. 2007). More recently, we identified that the modulation of SYP132 abundance at the PM during bacterial pathogenesis in Arabidopsis influences PR1 secretion (Baena et al. 2022). This defense-related secretory pathway involves SYP132 binding within SNARE core complexes with R-SNAREs VAMP721 and VAMP722 (Xia et al. 2019; Kim et al. 2021; Baena et al. 2022). SYP132 abundance and functions at the PM are hormone regulated (Xia et al. 2019) impacting plant growth, stomatal regulation, and pathogen defense (Enami et al. 2009; Xia et al. 2019, 2020; Kim et al. 2021; Baena et al. 2022, 2024). Beyond these studies, much detail of SYP132 secretory cargoes remains unknown, and this knowledge is vital to understand the how the SNARE coordinates plant growth and pathogen defense.

To resolve the full breadth of the Arabidopsis secretory proteome associated with SYP132, here we carried out advanced quantitative MS involving tandem mass tagging (TMT-MS) of the apoplast flush by comparing samples from *syp121/syp122* double mutant plants against wild-type Arabidopsis. Tracking native proteins using immunoblot, we found that SYP132 is sufficient for basal secretion when both SYP121 and SYP122 are not expressed in the plant. Notably, the increase in expression of genes associated with SA signaling in the double mutant plants corresponds to increased SYP132 expression and abundance at the PM. Using SYP132 overexpression or pathogen stress as a deterrent of the SNARE abundance at the PM, we identified that SYP132 influences the apoplastic accumulation of several secreted cargoes, notably of proteins associated with salicylic acid (SA) and jasmonic acid (JA) signaling, including the pathogenesis-related proteins PR1, PR2, and PR5. We propose therefore that secretory traffic associated with the SNARE SYP132 impacts plant growth and pathogen defense pathways.

## Results

### Arabidopsis SYP132 is sufficient for basal secretory traffic and growth

**Figure 1.**
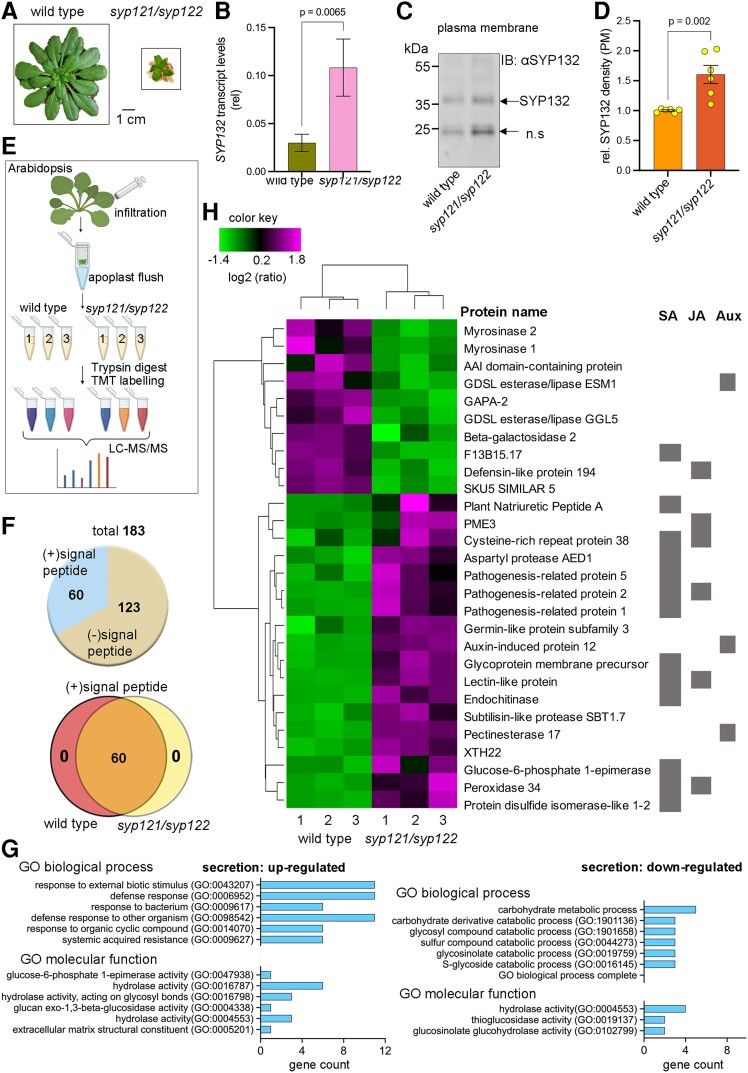
Analysis of leaf apoplast secreted proteins associated with trafficking SNARE SYP132 using wild type and *syp121/syp122* mutant Arabidopsis (c.f. Supplementary Tables S1 and S2, and Fig. S1). **A)** Images (representative) of 4-wk-old soil-grown *A. thaliana* wild-type and *syp121/syp122* mutant plants used in the experiments. Scale bar = 1 cm (for both panels) (c.f. Supplementary Fig. S1A for images from corresponding experiments with plate-grown plants for flood inoculation with bacterial pathogens *Pst*). Images were digitally extracted for comparison. **B)***SYP132* transcript levels in wild type and *syp121/syp122* mutant Arabidopsis leaf tissue relative to 18S using RT-qPCR with gene-specific primers (c.f. Supplementary Table S1). Data are mean ± Se (*n* = 3). Statistical significance using 2-tailed Mann–Whitney *t* test is indicated. **C)** Immunoblots (representative) of purified PM proteins derived from wild-type and *syp121/syp122* mutant Arabidopsis leaves. Proteins were resolved using SDS-PAGE (c.f. Supplementary Fig. S1B for corresponding immunoblots detecting lumen-binding protein BiP [∼74 kDa], a marker for IMs, and Coomassie-stained membranes for total protein). Purity of the PM fractions using BiP bands as reference was estimated as >99%. Native SYP132 proteins (∼35 kDa) were detected using anti-SYP132 antibodies. A low molecular weight nonspecific band detected is marked n.s. Black lines (left) indicate position of molecular mass markers, and black arrows (right) point to expected band positions. **D)** Mean ± Se (*n* = 6) SYP132 protein density in PM fractions of wild type and *syp121/syp122* mutant Arabidopsis leaf tissue. Values are obtained from densitometric analysis of immunoblots and normalized to total protein in each lane detected using Coomassie stain. Statistical significance using 2-tailed Mann–Whitney *t* test is indicated. **E)** Workflow for TMT-MS analysis of Arabidopsis secretory proteome. Apoplast flush was collected from wild-type and *syp121/syp122* mutant Arabidopsis leaves following infiltration with 10 mm MgCl_2_ buffer using centrifugation (Grefen et al. 2015; Xia et al. 2019; Baena et al. 2022). Samples from 3 independent experiments were trypsin digested, labeled with reagents from TMT-11plex isobaric mass tagging kit (Thermo Scientific), and detected using electrospray ionization MS. Acquired MS/MS spectra were analyzed for protein identification and quantification using Proteome Discoverer software 3.0 (Thermo Scientific) using the MSPepSearch node. For quantitative changes, protein ratios for cargoes detected in wild-type vs *syp121/syp122* mutant plants were computed (c.f. Supplementary Table S2). **F)** Venn diagrams depicting distribution of secreted cargo (+)/(−) signal peptides identified in wild type and *syp121/syp122* mutant plants (top) and the overlap of cargoes with signal peptides detected in wild type and *syp121/syp122* mutant Arabidopsis (bottom). Cargo proteins with signal peptides are considered as secretory vesicle cargoes. **G)** Bar graphs showing the assignment of GO terms to cargoes that undergo secretory vesicle trafficking, based on GO annotation categories, including molecular and biological functions for cargoes that show upregulation (left) and downregulation (right) in abundance in the apoplast flush. Analysis was performed using the PANTHER database program (www.pantherdb.org). **H)** Heatmap depicting hierarchical clustering of secretory cargoes detected in *syp121/syp122* mutant compared against wild-type Arabidopsis using TMT-MS analysis. Cargoes with statistically significant differences in TMT ratio analyzed by ANOVA are shown (*P* ≤ 0.05). Cargoes associated with hormones SA, JA, and auxin (Aux) signals are indicated (gray bars). Data are from 3 independent experiments.

Homozygous *syp132* mutant Arabidopsis are not viable due to defects in cytokinesis (Park et al. 2018); however, fully developed Arabidopsis *syp121/syp122* double mutant plants complete their life cycle and produce seed (Assaad et al. 2004; Zhang et al. 2008). To elucidate the potential role(s) of SYP132 in the secretory traffic to the apoplast, we used the *syp121-1 (=pen1)/syp122-1* mutant Arabidopsis. In the absence of both SYP121 and SYP122, secretory vesicle traffic in the *syp121/syp122* Arabidopsis is primarily attributed to the SNARE SYP132 (Waghmare et al. 2018; Rubiato et al. 2022). The *syp121-1/syp122-1* plants exhibit severely stunted growth (Fig. 1A) (Zhang et al. 2007). Syntaxin double mutants of 3 additional alleles of *syp121* each with a T-DNA insertion allele of *syp122* were characterized in the past, all exhibiting identical phenotypes consistent with the lack of SYP121 and SYP122 (Zhang et al. 2007). The *syp121-1/syp122-1* mutant plant line is the most commonly used tool in studies to characterize SYP121 and SYP122 functions (Zhang et al. 2008).

Using *SYP132-*specific primers (Supplementary Table S1) in reverse transcription quantitative PCR (RT-qPCR) analysis (Baena et al. 2022), we found that *SYP132* transcript level in 4-wk-old *syp121/syp122* mutant plants is approximately 2-fold higher compared to wild-type plants (Fig. 1B). Using immunoblot analysis with SYP132-specific antibodies (Baena et al. 2022), we found that SYP132 protein abundance at the PM in double mutants was 2-fold higher compared to the wild-type plants (Fig. 1, C and D, Supplementary Fig. S1B). Thus, SYP132-assisted secretion, we expected, would predominate in the *syp121/syp122* mutant plants.

To examine the influence of SYP132-assisted vesicle traffic, we carried out TMT-MS analysis (Li et al. 2020) of extracellular secreted proteins in leaf tissue. Apoplast flush samples collected from 4-wk-old soil-grown Arabidopsis leaves (Grefen et al. 2015; Xia et al. 2019) were trypsin digested, labeled with 11-plex TMT reagents (Guan et al. 2019) and analyzed using electrospray ionization MS (Fig. 1E) (see Materials and methods). TMT reagents are isobaric and can be used to label and analyze tryptic peptides for concurrent identification and relative quantification of proteins in different samples using MS (Li et al. 2020). A total of 183 proteins were identified in the apoplast flush (Supplementary Table S2). Of these, 60 cargoes contained the secretory signal peptide essential for SNARE-assisted vesicle traffic and the remaining cargoes were attributed to constitutive secretory pathways (Waghmare et al. 2018).

We expected that SYP121 and SYP122 secretory cargoes would be absent in *syp121/syp122* plants. Yet, all of the 60 SNARE-assisted secretory cargoes occurred in both wild-type and *syp121/syp122* plants (Fig. 1F). These data suggested that in the absence of both SYP121 and SYP122, the SNARE SYP132 facilitates basal secretion (Karnahl et al. 2018; Park et al. 2018; Waghmare et al. 2018). Ratiometric TMT-MS analysis was carried out to evaluate significant differences in relative abundance of each cargo in the proteome. Differentially abundant cargoes were identified as those which occur within a threshold of log_2_ (fold change) ≥0.5 or ≤−0.5 and discount background (Supplementary Table S2). The expectation was that SYP132-dependent cargoes would have altered abundance in the apoplast flush of *syp121/syp122* plants relative to the wild type. Remarkably, we found 28 secretory cargoes with significant changes in abundance in *syp121/syp122* mutant plants relative to the wild type. We did not detect GLUCOSE-6-PHOSPHATE DEHYDROGENASE, a reporter for cytosol leak in the apoplast (Alexandersson et al. 2013) (Supplementary Table S2). Two additional cargoes that generally occur in the cytosol, MALATE DEHYDROGENASE 1 (MDH, AT1G04410) and CATALASE 3 (CAT3, AT1G20620) (Alexandersson et al. 2013), were detected at low and constant levels. Regardless, TMT-MS ratios of these proteins in *syp121/syp122* mutant relative to the control (wild-type) plants indicated that contaminant background remained uniform across all samples. Therefore, we are confident that findings from ratiometric analysis were not influenced.

Gene Ontology (GO) biological process classification of the differentially regulated cargoes showed that secretion of 18 cargo proteins associated with pathogen defense, biotic stimuli, and system-acquired resistance was upregulated in the *syp121/syp122* mutants (Fig. 1G, Supplementary Table S2), and these cargoes had functions such as hydrolase or glucose-6-phosphate 1-epimerase activities and extracellular matrix constitution. In contrast, the 10 secretory cargoes with reduced abundance in the apoplast flush were involved in carbohydrate metabolism and catabolic processes important for plant growth including hydrolase, thioglucosidase, and glucosinolate glucohydrolase activities (Fig. 1G, Supplementary Table S2).

### SYP132 influences secretion of cargoes associated with pathogen defense and cell wall plasticity

To visualize the quantitative changes in secretory proteome, we measured relative abundance patterns of differentially regulated cargoes with secretory signal peptides in the apoplast flush from 3 replicates each from wild-type and *syp121/syp122* mutant plants (Fig. 1H). We expected that comparing the apoplast proteome from *syp121/syp122* mutant plants with that of wild-type Arabidopsis would help determine if the SNARE SYP132 influences the secretion of defense-related cargoes. TMT-MS data were subjected to hierarchical clustering with a Pearson correlation. These analyses revealed opposite trends for secretory proteomes derived from wild-type plants compared against the *syp121/syp122* mutant (Fig. 1H).

Arabidopsis *syp121/syp122* mutants are unable to block fungal ingress (Rubiato et al. 2022), and it is well established that penetration resistance relies on SYP121-mediated pathways (Lipka et al. 2005; Zhang et al. 2007). The *syp121/syp122* plants constitutively express components of the SA signaling pathway, and levels of SA are high (Zhang et al. 2007). The dwarfed rosette phenotype and autoimmune characteristics are attributed to the elevated SA in *syp121/syp122* plants. Prior to the onset of necrosis, the *syp121/syp122* mutants exhibit a short period of normal growth and are visually indistinguishable from wild-type plants (Rubiato et al. 2022). Bacterial pathogen challenge triggered significant necrosis in the mutant plants (Supplementary Fig. S1A), and these observations align with previous studies indicating that SA signaling pathways contribute to bacterial pathogen defense (Zhang et al. 2007). The spontaneous development of severe necrotic lesions and cell death in the leaf tissue makes it difficult to reliably assess bacterial pathogen proliferation in the *syp121/syp122* plants (Assaad et al. 2004; Zhang et al. 2007, 2008). Additional mutations that silence SA signaling in the *syp121/syp122* mutants can partially overcome necrosis and suggest altered susceptibility to bacterial pathogens (Zhang et al. 2007, 2008). Thus, it is likely that defense-related vesicle trafficking pathways are active independent of SYP121 and SYP122.

The accumulation of cargoes for pathogen defense involving SA and/or JA signaling was pronounced in *syp121/syp122* mutant plants compared to the wild type (Fig. 1H). Notably, the abundance of PECTIN METHYLESTERASE 3 (PME3), ENDOCHINTINASE (CHI), PLANT NATRIURETIC PEPTIDE-A (PNP-A), and PATHOGENESIS-RELATED PROTEIN (PR) family proteins increased in *syp121/syp122* mutant plants compared to the wild type (Fig. 1H, Supplementary Table S2). Both PME and CHI are cell wall modification enzymes that influence bacterial and fungal pathogen defense (Bethke et al. 2013; Xue and Yi 2018); PNP-A and PR proteins are involved in plant responses to biotic and abiotic challenges (van Loon et al. 2006; Han et al. 2023). Taken together, these data suggest that in *syp121/syp122* mutant plants, SYP132 may play a central role in influencing secretory traffic of cargoes associated with SA signaling and pathogen defense.

### SYP132 influences secretion of pathogenesis-related PR1, PR2, and PR5 proteins to the apoplast

To evaluate the role of SYP132 in secretory vesicle traffic to the apoplast, TMT-MS analysis was carried out as before, this time to analyze apoplast flush proteins from SYP132-OX Arabidopsis transgenic lines overexpressing the SNARE (35S:RFP-SYP132; Xia et al. 2019) (see Materials and methods) with the expectation that secretory traffic associated with this SNARE is enhanced in these plants. SYP132-OX line 1 and line 2 used in the study have similar physiology and growth phenotypes (Xia et al. 2019; Baena et al. 2022) and exhibit qualitatively equivalent secretory proteome profiles based on label-free MS analysis (Supplementary Table S3, *N* = 4 independent experiments).

**Figure 2.**
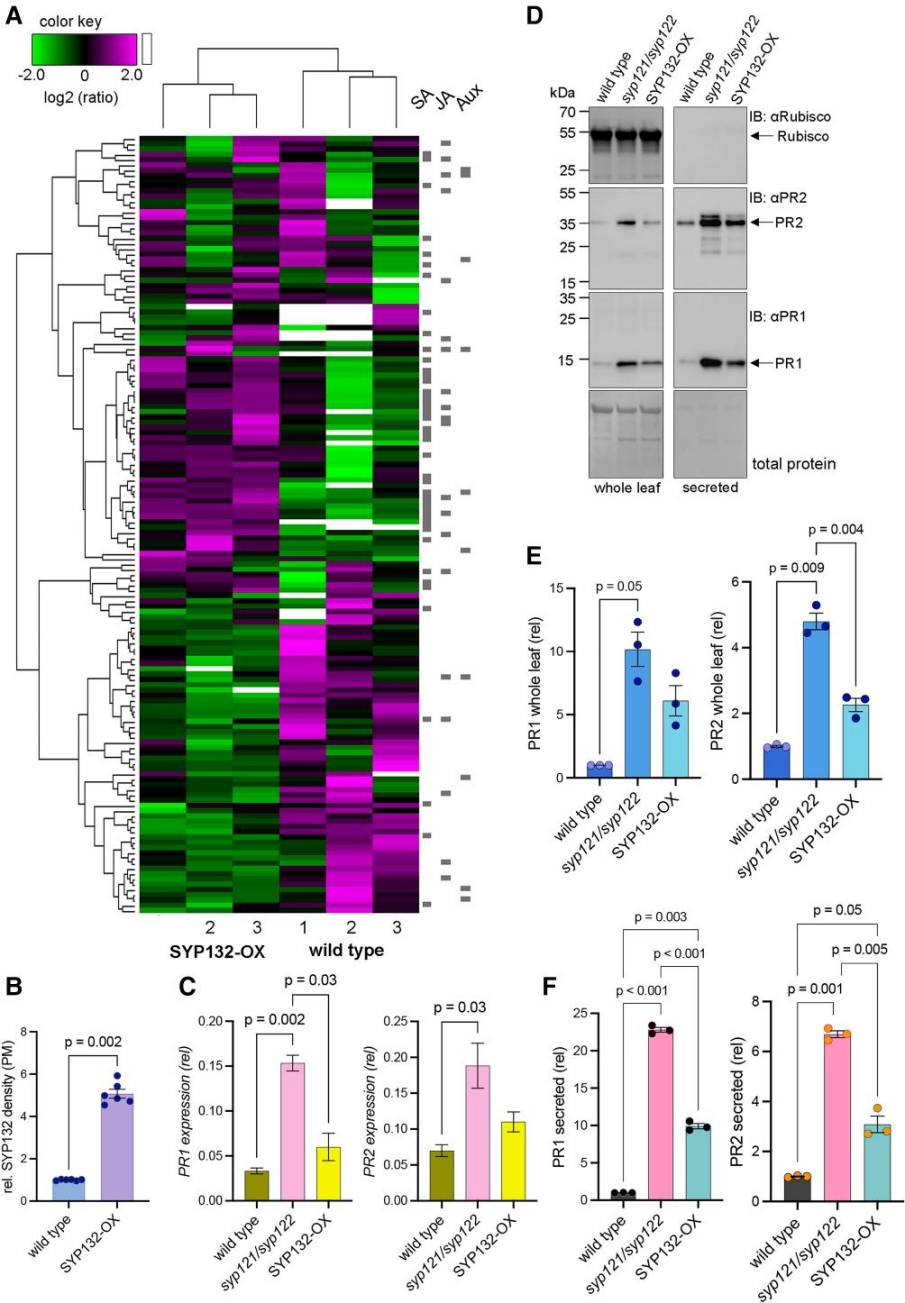
SNARE SYP132 influences secretion of cargoes associated with SA and JA signaling including the pathogenesis-related PR1 and PR2 proteins (c.f. Supplementary Tables S1, S4, and S5, Fig. 1C, Supplementary Figs. S2 and S3A). **A)** Heatmap depicting hierarchical clustering of secretory cargoes detected in SYP132-OX (35S:RFP-SYP132; Baena et al. 2022) Arabidopsis compared against wild-type plants using TMT-MS analysis. Cargoes associated with hormones SA, JA, and auxin (Aux) signaling are indicated (gray bars). Data are from 3 independent experiments (c.f. see Supplementary Tables S4 and S5). **B)** Mean ± Se (*n* = 6), total SYP132 protein density (native SYP132 + RFP-SYP132) in the PM fractions from SYP132-OX plants relative to wild type. Values were obtained from densitometric analysis of immunoblots, normalized against total protein in each lane detected using Coomassie stain (Fig. 1C, Supplementary Fig. S3A). Statistical significance determined using 2-tailed Mann–Whitney *t* test is indicated. **C)***PR1* and *PR2* expression in wild-type, *syp121/syp122* mutant, and SYP132-OX Arabidopsis leaf tissue relative to 18S using RT-qPCR with gene-specific primers (Supplementary Table S1). Expression of additional defense-related genes, *PME3*, *ENDOCITINASE*, and as control, *EXPANSIN-LIKE A1*, was determined (Supplementary Fig. S2). Data are mean ± Se (*n* = 3). Statistical significance using Brown–Forsythe and Welch ANOVA as indicated. **D)** Immunoblots (representative) of proteins in wild type, *syp121/syp122* mutant, and SYP132-OX Arabidopsis, whole leaf tissue (left panels), and apoplast flush (secreted, right panels). Proteins were resolved using SDS-PAGE. Native PR1 (∼15 kDa) and PR2 (∼34 kDa) proteins were detected using anti-PR1 and anti-PR2 antibodies, respectively. Rubisco heavy chain protein (∼53 kDa), marking cell interior (Gupta et al. 2011), was detected using anti-Rubisco antibodies. Purity of apoplast flush was estimated as >99% using Rubisco band as reference. Coomassie-stained membranes detect total protein. Black lines (left) indicate positions of molecular mass markers, and black arrows (right) indicate expected band positions. **E and F)** Mean ± Se (*n* = 3) PR1 and PR2 abundance in wild type, *syp121/syp122* mutant, and SYP132-OX Arabidopsis whole leaf **E)** and secreted apoplast flush **F)**, relative to wild-type Arabidopsis. Values from wild type, *syp121/syp122* mutant, and SYP132-OX plants were obtained from densitometric analysis of immunoblots and normalized to total protein in each lane detected using Coomassie stain. Statistical significance using Brown–Forsythe and Welch ANOVA as indicated.

Apoplast flush samples from the SYP132-OX plants were compared against wild-type plants (Fig. 2A). The SYP132-OX plants have approximately 5-folds more SNARE abundance (native SYP132 + RFP-SYP132) at the PM compared to wild-type plants (Fig. 2B) (Baena et al. 2022). Contaminant background was uniformly low across samples and therefore of no significance to ratiometric analysis. The proteins GLUCOSE-6-PHOSPHATE DEHYDROGENASE and CATALASE 3 that are highly abundant in the cytosol were not detected. TMT-MS data were subjected to hierarchical clustering with a Pearson correlation to visualize quantitative changes in abundance of the secretory cargoes and suggested that majority of the cargoes upregulated in the SYP132-OX compared to the wild-type Arabidopsis were involved in SA and JA signaling pathways (Fig. 2A, Supplementary Table S4). To evaluate specificity of SYP132, we tracked the 18 secreted cargoes significantly upregulated in *syp121/syp122* mutant relative to the wild type (Fig. 1H) against cargoes that were highly abundant in the SYP132-OX plants (Supplementary Tables S2 and S5). We found 12 cargoes secreted to be highly abundant in both the plant lines (Supplementary Fig. S2A and Table S6). These 12 cargoes were therefore deemed most likely to be secreted via SYP132-specific vesicle traffic, including the PR1, PR2, and PR5 proteins, PLANT NATRIURETIC PEPTIDE-A (PNP-A, AT2G18660), and ENDOCHITINASE (CHI, AT2G43570) (Figs. 1H and 2A, Supplementary Tables S2, S4, and S6). Overexpression and accumulation of PR1 in the apoplast promote resistance against bacterial pathogens in several plant species (Kalde et al. 2007; Arabi et al. 2020; Anisimova et al. 2021; Pecenkova et al. 2022; Han et al. 2023). SYP132 facilitates PR1 secretion essential for resistance to bacterial infection (Kalde et al. 2007; Baena et al. 2022). Thus, our observations indicate that SYP132 might be involved in secretion of additional defense-related cargoes.

In parallel, we evaluated transcript levels of representative genes for cargoes that we identified as upregulated in the secretory proteomes of both *syp121/syp122* and SYP132-OX plants. Gene expression was measured by RT-qPCR using gene-specific primers (see Materials and methods). We found that *PR1*, *PR2*, *PME3*, and *CHI* transcripts were similar in wild-type and SYP132-OX plants but were significantly higher in *syp121/syp122* mutants (Fig. 2C, Supplementary Fig. S2, B to D). Thus, increased expression does not preclude increased secretion to the apoplast.

Corresponding to the MS, immunoblot analysis of 2 defense-related secretory cargoes PR1 and PR2 was carried out using protein-specific antibodies to detect total PR1 and PR2 protein levels in whole leaf tissue (Fig. 2, D and E) and in the apoplast flush for the secreted protein pool (Fig. 2, D and F). The purity of the apoplast flush was determined as >99% using Rubisco, a highly abundant protein that occurs within the cell, as a reference. We found that secreted PR1 and PR2 levels to be significantly higher in both *syp121/syp122* mutant SYP132-OX leaves compared to the wild-type plants, although the abundance of these proteins in the whole plant was significantly elevated only in the *syp121/syp122* mutants. Thus, it is likely that increased SYP132 density promotes secretory traffic of PR1 and PR2 proteins at the PM.

**Figure 3.**
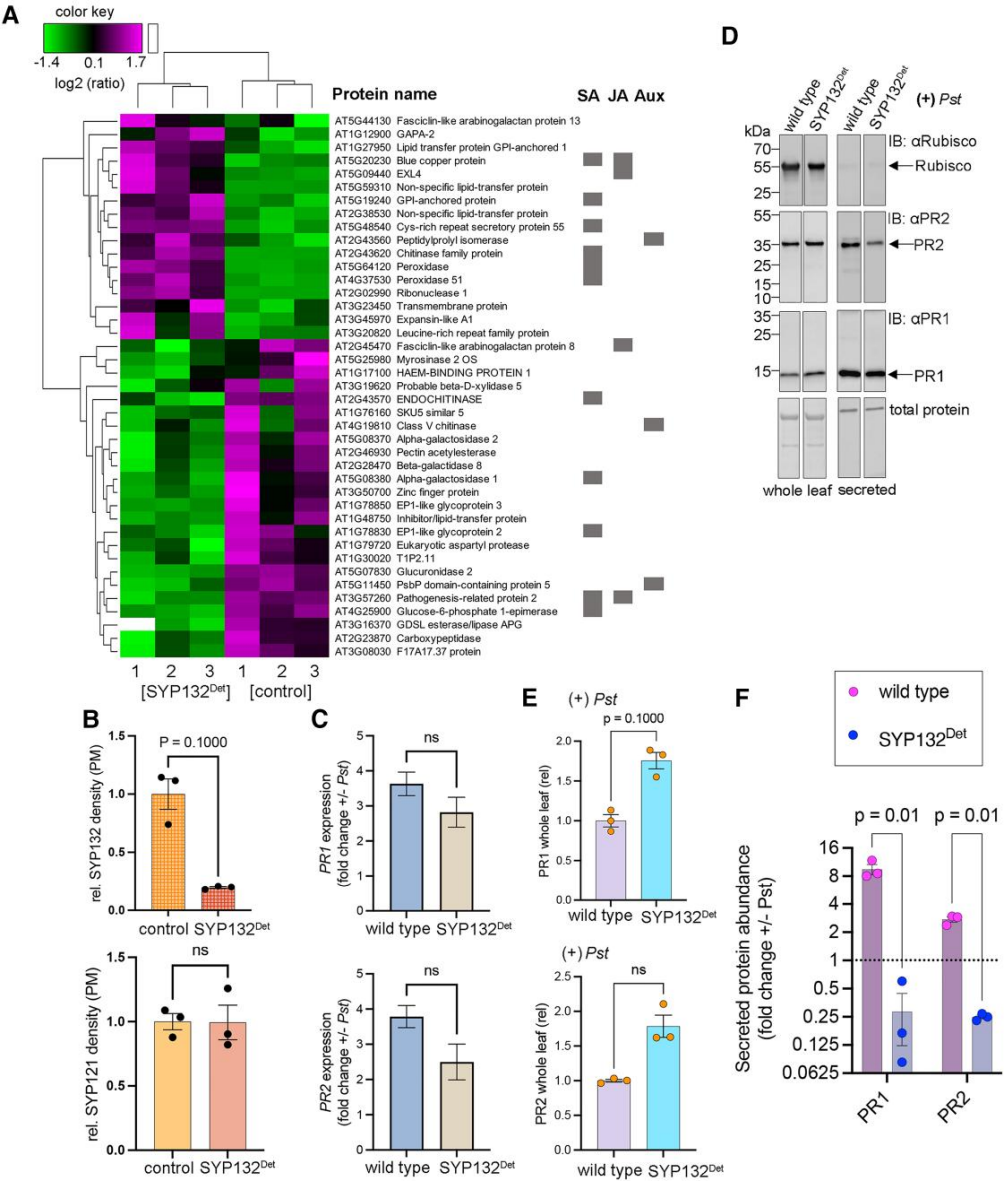
Interfering with SYP132 affects the secretion of cargoes associated with SA and JA signaling including pathogenesis-related PR1 and PR2 proteins (c.f. Supplementary Tables S1 and S5, and Fig. S3). **A)** Heatmap depicting hierarchical clustering of secretory cargoes using TMT-MS analysis detected in SYP132-OX (control, 35S:RFP-SYP132) compared against SYP132^Deterrent^ (SYP132^Det^, [+]*Pst*) Arabidopsis. Cargoes with statistically significant differences for TMT ratios analyzed by ANOVA (*P* ≤ 0.05) are shown. Cargoes associated with hormones SA, JA, and auxin (Aux) signaling are indicated (gray bars on the right) (c.f. Supplementary Table S5). Data are from 3 independent experiments. **B)** Mean ± Se (*n* = 3), total SYP132 (native SYP132 + RFP-SYP132, top panel), and SYP121 (bottom panel) protein abundance in the PM fractions from SYP132^Det^ (SYP132-OX [+]*Pst*) plants relative to control (SYP132-OX). Values were obtained from densitometric analysis of immunoblots, normalized against total protein in each lane detected using Coomassie stain (Supplementary Fig. S3A). Statistical significance determined using 2-tailed Mann–Whitney *t* test is indicated (*P* ≥ 0.05 was considered as “not significant”). **C)** Mean ± Se (*n* = 3) fold change in expression of *PR1* (top panel) and *PR2* (top panel) in Arabidopsis wild type and SYP132^Det^ leaf tissue treated with bacterial pathogens (*Pst* compared against buffer, 10 mm MgCl_2_). Data are 2^ΔΔCt^ relative to 18S using RT-qPCR with gene-specific primers (c.f. Supplementary Table S1). Statistical significance using Brown–Forsythe and Welch ANOVA as indicated (*P* ≥ 0.05 was considered as “not significant”). Expression of an additional defense-related gene *PME3* was determined (c.f. Supplementary Fig. S3F). **D)** Immunoblots (representative) of proteins in whole leaf tissue (left panels) and secreted (apoplast flush, right panels) from wild type and SYP132^Det^ Arabidopsis infected with bacterial pathogens ([+]*Pst*). Proteins were resolved using SDS-PAGE. Native PR1 (∼15 kDa) and PR2 (∼34 kDa) proteins were detected using anti-PR1 and anti-PR2 antibodies, respectively. Rubisco heavy chain protein (∼53 kDa) marking IMs was detected using anti-Rubisco antibodies to determine purity of apoplast flush (estimated >99%). Coomassie-stained membranes detect total protein. Black lines (left) indicate positions of molecular mass markers, and black arrows (right) indicate expected band positions. **E)** Mean ± Se (*n* = 3), PR1, and PR2 abundance in whole leaf tissue of SYP132^Det^ plants relative to wild-type Arabidopsis following bacterial pathogen infection ([+]*Pst*). Values were obtained from densitometric analysis of immunoblots (**D**, left panels) and normalized to total protein in each lane detected using Coomassie stain. Statistical significance determined using two-tailed Mann Whitney t test is indicated (*P* ≥ 0.05 was considered as “not significant”). **F)** Mean ± Se (*n* = 3), fold change secreted PR1 (top panel), and PR2 (bottom panel) protein abundance in wild-type and SYP132^Det^ plants following bacterial pathogen (+*Pst*) infection, relative to plants treated with buffer (10 mm MgCl_2_). Values were obtained from densitometric analysis of immunoblots normalized to total protein in each lane detected using Coomassie stain. Statistical significance determined using *t* test with Welch correction on each row, and Holm–Sidak's multiple comparisons test is indicated (*P* ≥ 0.05 was considered as “not significant”).

When SYP132 is overexpressed, its redistribution to internal membranes (IMs) under stress reduces SNARE density and is selectively deterrent for its functions at the PM (Baena et al. 2022, 2024). We made use of this behavior as tool for suppressing SYP132-mediated secretion at the PM. SYP132-OX Arabidopsis plants challenged with bacterial pathogens *Pseudomonas syringae* pv. tomato DC3000 (*Pst*) (see Materials and methods) were used in the analysis and are referred to as SYP132^Det^ (SYP132^Deterrent^) to highlight that they have reduced SNARE abundance due to disease stress. SYP132 protein levels at the PM in SYP132^Det^ plants were >5-fold lower compared the control (SYP132-OX, untreated) plants (Fig. 3B, Supplementary Fig. S3A). We also analyzed transcript levels of homologous SNAREs *SYP121* and *SYP122* in SYP132-OX plants and found them to be similar to wild-type Arabidopsis (Supplementary Fig. S3, B and C). There was no change in SYP121 protein density at the PM in plants compared to the control (Supplementary Fig. S3A, Fig. 3B). Thus, changes in SYP132 abundance do not affect homologous SNAREs.

To evaluate the impact on secretory traffic to the apoplast, TMT-MS analysis (see Materials and methods) of apoplast flush samples from the pathogen-infected SYP132^Det^ leaves was compared against SYP132-OX leaves, treated with buffer as control. A small number of cargoes were upregulated in the SYP132^Det^ apoplast, for example EXPANSIN-LIKE A1 (EXLA1) proteins thought to be important for cell wall plasticity, but not yet fully characterized in plants (Sampedro and Cosgrove 2005). The expectation was that defense-related cargoes that rely on the SNARE SYP132 for secretion to the apoplast should be reduced in the SYP132^Det^ relative to the control. We subjected TMT-MS data from 3 replicate proteomes from the plant lines to hierarchical clustering with a Pearson correlation for cargoes that were significantly altered in the apoplast flush. Secretory proteomes (3 replicates) from SYP132^Det^ had opposing trends relative to the control (Fig. 3A). It was found that the abundance of cargoes associated with defense-hormone signaling, including PR2, CHI, and GLUCOSE-6-PHOSPHATE 1-EPIMERASE proteins to be significantly downregulated in SYP132^Det^ plants (Fig. 3A, Supplementary Table S5). PR1 secretion in response to *Pst*, it appeared, was blocked in these plant lines (Fig. 3D, Supplementary Table S5) as pathogen infection did not promote the accumulation of these proteins in the apoplast (Baena et al. 2022). These data suggest that both PR1 and PR2 are secreted into the apoplast in a SYP132-dependent manner.

It cannot be completely ruled out that changes in gene expression contribute to the changes in cargo abundance in the apoplast. To examine this possibility, we measured fold change in *PR1* and *PR2* transcripts together with levels of *PME* using RT-qPCR (see Materials and methods) following pathogen infection (+/−*Pst*) and found no significant differences between wild type and SYP132^Det^ Arabidopsis (Fig. 3C, Supplementary Fig. S3D). Corresponding immunoblot analysis of whole leaf tissue showed approximately a 1.5-fold increase in PR1 and PR2 protein levels in both wild-type and SYP132^Det^ plants following *Pst* infection (Fig. 3, D and E). Analysis was also carried out using immunoblot to measure fold change in PR1 and PR2 protein abundance in the apoplast following pathogen infection (+/−*Pst*) in wild-type and SYP132^Det^ plants. We observed significant increase in the abundance of total PR1 (8-fold) and PR2 (3-fold) in wild-type plants following pathogen infection, even so levels of secreted PR1 and PR2 were significantly reduced in the SYP132^Det^ plants (Fig. 3F). Taken together, these data demonstrate that deterring SYP132 influences secretory traffic of defense-related cargoes, including the PR1 and PR2 independent of changes in their expression.

## Discussion

Plant–pathogen interactions activate defense-hormone signaling and transcriptional reprograming impacting plant growth, development, and immunity (Jones and Dangl 2006; Spoel and Dong 2024). Pathogen defense mechanisms rely on the biosynthesis of immunity-related molecules such as signaling proteins, reactive oxygen species (ROS), phytoalexins, phenolic compounds, and pathogenesis-related (PR) proteins, several of which are secreted and accumulate within the apoplast to modify the cell wall and to resist pathogenesis (Collins et al. 2003; Kwon et al. 2008; Meyer et al. 2009; Yun and Kwon 2017; Zarsky 2022; Yun et al. 2023). Our analysis demonstrates the vital importance of the SNARE SYP132 for basal secretion that underpins pathogen immunity. We have found that the leaf secretory proteomes of wild-type and *syp121/syp122* mutant Arabidopsis are identical, but the abundance of cargoes that associate defense-hormone signaling is significantly upregulated. Comparing secretory proteomes from wild-type and SYP132-overexpressing Arabidopsis, and using SYP132^Det^ plants with suppressed SNARE function, we identified the pathogenesis-related PR2 and PR5 proteins as potential SYP132-dependent secretory cargoes. The proteomics data together with analysis of expression using RT-qPCR and immunoblot assays to track native protein suggests that SYP132 impacts SA defense signaling and cell wall plasticity impacting pathogen immunity and basal growth in plants.

### SYP132 facilitates vesicle traffic associated with basal growth

The SNAREs SYP121, SYP122, and SYP132 are the primary contributors to secretory traffic at the PM in Arabidopsis (Enami et al. 2009). In general, the transcript levels of *SYP121* and *SYP122* are relatively highly abundant among Qa-SNAREs (Xia et al. 2019) and the respective proteins share >65% sequence similarity (Assaad et al. 2001; Bassham and Blatt 2008). We have now found that *SYP132* expression and the abundance of SYP132 protein at the PM are significantly increased in *syp121/syp122* plants compared to the wild type (Fig. 1, B to D). Corresponding differences in the accumulation of secreted proteins between wild-type and *syp121/syp122* plants (Fig. 1, F to H) could be explained, at least in part, by the increased expression of SYP132 (Fig. 1, B to D). Remarkably, subsets of secreted cargo proteins detected in wild-type and *syp121/syp122* mutant Arabidopsis were identical, albeit differences in their abundance. Thus, even in the absence of the 2 major SNAREs, SYP132 must support a level of basal secretion (Fig. 1F). Since *syp121/syp122* plants are viable, it may also be that SYP132 shares partial functional redundancy with the SNAREs SYP121 and SYP122 facilitating secretory vesicle traffic sufficient for basal growth. Changes in SYP132 abundance at the PM is likely to have a direct consequence on the trafficking of cargoes associated with SA and JA signaling. Our observation that native SYP132 abundance at the PM is significantly elevated in the *syp121/syp122* mutant (Fig. 1, B to D) supports this hypothesis. However, it does not rule out if additional Qa-SNAREs or non-SNARE pathways also contribute to the secretory traffic and may be conscripted into trafficking activities in the double mutants. Thus, more knowledge is essential to understand role of individual SNAREs to resolve the regulation of complex trafficking events in plants.

### SYP132 is directly involved in bacterial pathogen immunity

Of interest, the secretory proteomes derived from wild type, *syp121-1*, and *syp122-1* Arabidopsis suggest that the SNAREs SYP121 and SYP122 share a significant functional overlap (Waghmare et al. 2018). Even so, these SNAREs also facilitate secretion of distinct cargo subsets. For instance, we know that majority of SYP121- and SYP122-dependent secretory cargoes are involved in carbohydrate metabolism, lipid metabolism, seed storage, oxidative and cell wall composition (Waghmare et al. 2018). Preferential binding of SYP121 with the SNARE regulatory Sec1/Munc18 protein SEC11 (=KEULE) distinguishes it from SYP122 and impacts vegetative plant growth (Karnik et al. 2013, 2015). SYP121 specifically mediates vesicle traffic of aquaporin PIP2;7 (Hachez et al. 2014) and K^+^ channels (Sutter et al. 2006a, 2006b; Honsbein et al. 2009; Grefen et al. 2010; Grefen et al. 2015) at the PM, but it has no apparent role in PM H^+^-ATPase trafficking (Sutter et al. 2006a, 2006b; Xia et al. 2019). Additionally, selective binding of SYP121 with the K^+^ channels promotes channel gating and coordinates vesicle traffic with ion transport in plant cells (Honsbein et al. 2009; Grefen et al. 2015; Zhang et al. 2015, 2017, 2019; Karnik et al. 2017; Lefoulon et al. 2018).

SNAREs SYP121 and SYP122 mediated traffic provides pre- and postinvasive immunity against powdery mildew (*Blumeria graminis* f. sp. *hordei*) and filamentous fungal pathogens (Collins et al. 2003; Rubiato et al. 2022), including the release of exosomes to the outside of the cell (Meyer et al. 2009). In contrast, the *syp121-1*, but not the *syp122-1* mutants show a strong phenotype under drought stress (Eisenach et al. 2012). These and several other reports suggest that SYP121 and SYP122 originated in early land plants to perform highly specialized functions in abiotic stress resilience and in the immunity against fungal pathogens (Leyman et al. 1999; Sanderfoot et al. 2000; Sutter et al. 2006a, 2006b; Eisenach et al. 2012; Kanazawa et al. 2016; Park et al. 2018; Rubiato et al. 2022; Yun et al. 2023).

A partial redundancy between the SNAREs is cited as an explanation for the lack of strong phenotypes for *syp121-1* and *syp122*-1 mutants, although differences in growth and stomatal behaviors are clearly evident in these mutants under abiotic and biotic stress (Eisenach et al. 2012). Early studies did suggest that SYP121 and SYP122 are negative regulators of pathogen-inducible defense pathways associated with phytohormone signaling including SA and JA (Zhang et al. 2007), but these pathways are since thought to impact plant immunity through convergence and antagonism of different signals (Zhang et al. 2020). Indeed, SA levels in *syp121/syp122* double mutant plants are dramatically elevated (Zhang et al. 2008) resulting in necrosis and dwarfism (Assaad et al. 2004) (Fig. 1A, Supplementary Fig. S1A), and this necrotic phenotype can be delayed by introducing mutations to SA signaling genes (Zhang et al. 2007, 2008).

SNARE pathways in bacterial pathogen defense may exhibit specificity in function. We suggest that secretory traffic of cargoes associated with SA and JA signaling is influenced by SYP132. This hypothesis is supported by the increased abundance of secreted cargoes in SYP132-OX plants that associate with SA and JA signals (Fig. 2) and by the suppression of defense-related secretion in SYP132^Det^ plants (Fig. 3) that we found. However, the specificity of cargo selection in SYP132 vesicle traffic might also rely on additional components of the SYP132 SNARE complex machinery. For instance, it is likely that exocyst tethering complex and/or Rab GTPase RabA2a influence the specificity of vesicle trafficking pathways in fungal pathogen defense (Zarsky 2022; Nielsen 2024). During bacterial pathogenesis, a Synaptotagmin 5 (SYT5) protein regulates the assembly of SYP132 secretory SNARE complex with vesicle-localized R-SNAREs VAMP721 and VAMP722 (Kim et al. 2021). VAMP721/722 are involved in both fungal and bacterial pathogen defense, and they also bind other Qa-SNARE proteins (Kwon et al. 2008; Kim et al. 2021). SNARE SYP121 also forms ternary SNARE core complexes with VAMP721 and VAMP722 (Kwon et al. 2008; Ebine et al. 2011; Karnik et al. 2013). In addition, SYP121 binding with the VAMP727 isoform drives vesicle fusion at the PM. Yet, none of these secretory SNARE complexes involving SYP121 or SYP122 directly contribute to the secretion of antibacterial proteins (Kalde et al. 2007; Zhang et al. 2007; Kwon et al. 2020). The impact on bacterial pathogen defense in the *syp121/syp122* double mutant plants is therefore likely a consequence of increased SYP132 function, rather than the lack of SNAREs SYP121 and SYP122.

### SYP132 contributes to SA signaling pathways

SYP132 is evolutionarily the most ancient PM Qa-SNARE and occurs in relatively low abundance compared to the SYP121 and SYP122. *SYP132* expression is upregulated during bacterial pathogenesis (Baena et al. 2022) but not under salinity stress during which the SNARE promotes internalization from the PM (Baena et al. 2024). We found increased *SYP132* levels and higher density of the SNARE protein in the PM in *syp121/syp122* plants (Fig. 1, B to D). We have found that several cargoes associated with pathogen defense and SA signaling to be specifically upregulated in both *syp121/syp122* double mutant and in SYP132-OX plants (Supplementary Fig. S2A and Table S6). These cargoes include, but are not limited to, the pathogenesis-related PR isoforms PR1, PR2, and PR5 (Fig. 2, B to E, Supplementary Table S6). It is likely therefore that expression of the SNARE itself may be regulated by the SA defense signals at a transcriptional level together with other defense-related genes including *PR1* and *PR2* (Fig. 1B, Supplementary Fig. S2).

It cannot be fully ruled out if at least a part of the increased abundance of cargoes such as PR1 and PR2 in the apoplast is a direct consequence of changes in gene expression. In the SYP132-OX plants, secreted protein levels were about 10-fold higher for PR1 and almost 4-fold higher for PR2 proteins compared to wild-type plants (Fig. 2F), although wild type and SYP132-OX Arabidopsis express similar levels of *PR1* and *PR2* transcripts (Fig. 2C). *SYP132* transcripts were >10-fold higher in the SYP132-OX plants (Xia et al. 2019), and the SNARE protein at the PM is almost 5-fold higher compared to the wild-type plants (Fig. 2B). These findings align with the previously established role of SYP132 in PR1 secretion (Kalde et al. 2007; Baena et al. 2022) and support our hypothesis that elevated SYP132 abundance at the PM (Fig. 2B) promotes associated vesicle traffic. There is additional evidence to suggest that elevated gene expression (Fig. 2C, Supplementary Fig. S3, B to D) is not a prerequisite for increased in secretory traffic. Both *PR1* and *PR2* transcripts were almost 4 times higher in pathogen challenged wild-type and SYP132^Det^ plants (Fig. 3C). Yet secreted PR1 and PR2 levels in SYP132^Det^ plants following pathogen treatments were significantly reduced relative to healthy plants (Fig. 3F). These observations correspond to the reduced SYP132 density at the PM in these plants (Fig. 3B) following pathogen stress (Baena et al. 2022).

We expect that secretory traffic goes hand in hand with changes in gene regulation; therefore, some increase in whole leaf protein levels is expected mainly due to increased accumulation of secreted cargoes in the apoplast. Indeed, whole leaf protein levels were 5-fold higher for PR1 and about 2-fold more for PR2 in SYP132^Det^ Arabidopsis compared to the wild-type plants (Fig. 2, D and E). Should secretion be solely a function of total protein abundance, the fold change in secreted PR1 and PR2 in pathogen infected plants (+*Pst*) relative to healthy plants (−*Pst*) would be significantly higher in the plant lines. On the contrary, we found that fold change for secreted PR1 and PR2 (+/−) *Pst* was significantly reduced in SYP132^Det^ but not in the wild-type plants. Thus, changes in SYP132 density at the PM impact associated cargo secretion.

Among cargoes associated with SA defense signaling (Figs. 1 to 3, Supplementary Tables S2 and S4) are several PR isoforms expressed in Arabidopsis (PR1 to PR17). These proteins show diverse biochemical compositions and biological functions that mainly influence pathogen immunity (van Loon et al. 2006; Han et al. 2023). For instance, PR1 (AT2G14610) and PR2 (AT3G57260) inhibit microbial infection, PR2 encoding cell wall degrading β-1,3-glucanase enzyme that inhibits fungal pathogen invasion (Arabi et al. 2020; Anisimova et al. 2021). PR1 accumulation in the apoplast is mainly associated with SYP132 (Kalde et al. 2007; Baena et al. 2022), although it may be that PR1 is also trafficked via unconventional pathways independent of secretory vesicles (Pecenkova et al. 2022).

Other highly abundant cargoes in *syp121/syp122* secretory proteome associated with pathogen defense included GLUCOSE-6-PHOSPHATE 1-EPIMERASE enzyme (uncharacterized, AT4G25900), SA-induced legume LECTIN-LIKE PROTEIN (SAI-LLP1, AT5G03350), AUXIN INDUCED IN ROOT CULTURE protein (AIR12, AT3G07390), and PEROXIDASE (PRX34, AT3G49120). SAI-LLP1 is involved in SA-activated systemic acquired resistance (SAR) for defense against bacterial pathogens including the *P. syringae* pv. tomato (Breitenbach et al. 2014). Both AIR12 and PER34 affect apoplastic redox state; AIR12 is a monoheme cytochrome b protein that promotes accumulation of ROS species (superoxide, hydrogen peroxide) to regulate plant response to necrotrophic pathogens (Costa et al. 2015), and PRX34 promotes apoplast ROS burst to inhibit bacterial pathogen infection (Daudi et al. 2012). Thus, we propose a role for SYP132 in secretion of pathogenesis-related PR2 and also likely PR5 protein cargoes to the apoplast and suggest that the SNARE is essential for SA defense signaling in plants.

### SNARE SYP132 is at the center of plant responses to biotic and abiotic stress

SYP132 is important for plant growth and stress responses (Enami et al. 2009; Park et al. 2018; Xia et al. 2019). SYP132 activity is essential for basal growth and cytokinesis (Kalde et al. 2007; Karnahl et al. 2018; Park et al. 2018). Homozygous *syp132* mutants are embryo lethal (Park et al. 2018). SYP132-OX plants exhibit reduce growth, a characteristic that is attributed primarily to SYP132 role in promoting PM H^+^-ATPase internalization from the PM. SYP132 regulation of PM H^+^-ATPases influences auxin-dependent plant growth, stomatal aperture, and root gravitropic responses (Xia et al. 2019, 2020). SYP132 also promotes the accumulation of pathogenesis-related PR1 proteins in the apoplast following bacterial pathogen challenge (Kalde et al. 2007; Baena et al. 2022). SYP132^Det^ plants with limited SNARE function at the PM, we suggest, exhibit compromised postinfection immunity against bacterial pathogen *Pst* (Baena et al. 2022). Finally, in high salinity and osmotic stress, SYP132 coregulates AHA1 and aquaporin PIP2;1 density and functions at the PM impacting growth (Baena et al. 2024). SYP121 abundance in the PM remains unchanged in wild-type and SYP132-OX plants in both control and stressed conditions (Baena et al. 2024). There is no evidence to suggest that SYP121 (or SYP122) expression is regulated by bacterial pathogens; we did not observe any effect of SYP132 overexpression on *SYP121* and *SYP122* transcripts (Supplementary Fig. S3, A to C), and pathogen infection did not alter SYP121 density at the PM (Fig. 3B). We propose therefore that SNARE SYP132 and the regulation of its abundance at the PM govern multifactorial biotic and abiotic stress responses impacting plant growth, development, and immunity.

The negative association between defense and plant growth is well established (Huot et al. 2014). Divergent mechanisms that are not fully resolved underlie the growth–defense tradeoffs, including those which stem from hormone crosstalk and metabolic expenditure and mainly involve transcriptional and translational coregulation (Karasov et al. 2017). How the secretory machinery contributes to the tradeoff between growth and defense is an open question. Nonetheless, we can now begin to assemble some of the pieces. We found that fold change in secreted PR1 and PR2 is significantly lower in SYP132^Det^ plants compared to the wild type (Fig. 3, D and F), although both plant lines show similar increase in transcript levels of *PR1* and *PR2* (Fig. 3C). Therefore, we suggest that SYP132-dependent secretory traffic contributes to PR1 and PR2 accumulation in the apoplast and might therefore be a factor impacting growth vs immunity tradeoffs.

Autoimmune phenotypes are commonly attributed to elevated SA, which signals the activation of nucleotide-binding leucine-rich repeat receptors (NLRs), immune sensors which recognize pathogen effectors that activate programmed cell death responses (Horsefield et al. 2019; Wan et al. 2019; Wang et al. 2019a, 2019b). Even so, *syp121/syp122* plants exhibit basal growth and produce seed, and therefore, it can be expected that secretory proteome of these plants will reflect the impact of defense on growth. It may be that enhanced immunity associated with these secretory cargoes suppresses growth. We found that the accumulation of several cell wall modifying enzymes was enhanced in *syp121/syp122* and SYP132-OX plants (Figs. 1 and 2, Supplementary Tables S2 and S5) including ENDOCHITINASE (CHI, AT2G43570), SUBTILISIN-LIKE SERINE PROTEASE (SBT1.7, AT5G67360), pectin methylesterase (PME17, AT2G45220), and PECTIN METHYLESTERASE (PME3, AT3G14310). AtCHI functions in the defense against fungal and insect pathogens through the destruction of their chitin-containing cell walls and may have additional roles in water-deficit stress responses (Bray 2004); AtSBT1.7 affects pectin methylesterification impacting cell wall plasticity and seed germination (Ranocha et al. 2014); AtPME17 strongly contributes to pathogen-induced pectin methylesterase activity associated with JA defense signaling (Del Corpo et al. 2020); and AtPME3 is a ubiquitous cell wall pectin methylesterase affecting cell wall and seed germination (Guénin et al. 2017).

SA defense-related proteins, we have found, are upregulated in the SYP132-OX apoplast (Fig. 2), and the secretory proteome profile is similar to that of *syp121/syp122* mutant plants (Fig. 1). SYP132-OX plants exhibit stunted growth in normal conditions, mainly attributed to reduced H^+^-ATPase activity (Xia et al. 2019). Both SYP132-OX and *syp121/syp122* mutant plants have higher SYP132 abundance at the PM relative to the wild-type plants (Figs. 1D and 2B). Therefore, it may be that SA signaling cargoes inhibit growth in SYP132-OX plants. The overexpression of SYP132 influences SNARE availability and functions at the PM (Xia et al. 2019), an approach that has proven to be an important tool in studies with this unusual SNARE (Xia et al. 2019, 2020; Baena et al. 2022, 2024). Abundance of SYP132 is highly regulated through the control of *SYP132* transcripts and protein levels (Xia et al. 2020). SYP132 binding with the H^+^-ATPase AHA1 (Baena et al. 2024) is thought to promote rapid turnover of the SNARE proteins at the PM under stress; pathogen infection deters SYP132 function in plants overexpressing the SNARE (SYP132^Det^) (Baena et al. 2022, 2024), a characteristic that mimics the SNARE inhibitory iSNARE phenomenon (De Benedictis et al. 2013). Irrespective of the mechanism (Baena et al. 2022, 2024), we find that selectively deterring SYP132 does not alter SYP121 abundance at the PM (Fig. 3B) and suppresses the accumulation of defense-related cargoes in the apoplast of SYP132^Det^ plants (Fig. 3). Conversely, cell wall modifying enzymes were downregulated in both *syp121/syp122* and SYP132^Det^ plants (Figs. 1 and 3, Supplementary Table S6). Together with SA-induced necrosis, changes in cell wall plasticity might contribute to the increased susceptibility to pathogen infection in both these plant lines (Supplementary Fig. S1A) (Zhang et al. 2007; Baena et al. 2022).

**Figure 4.**
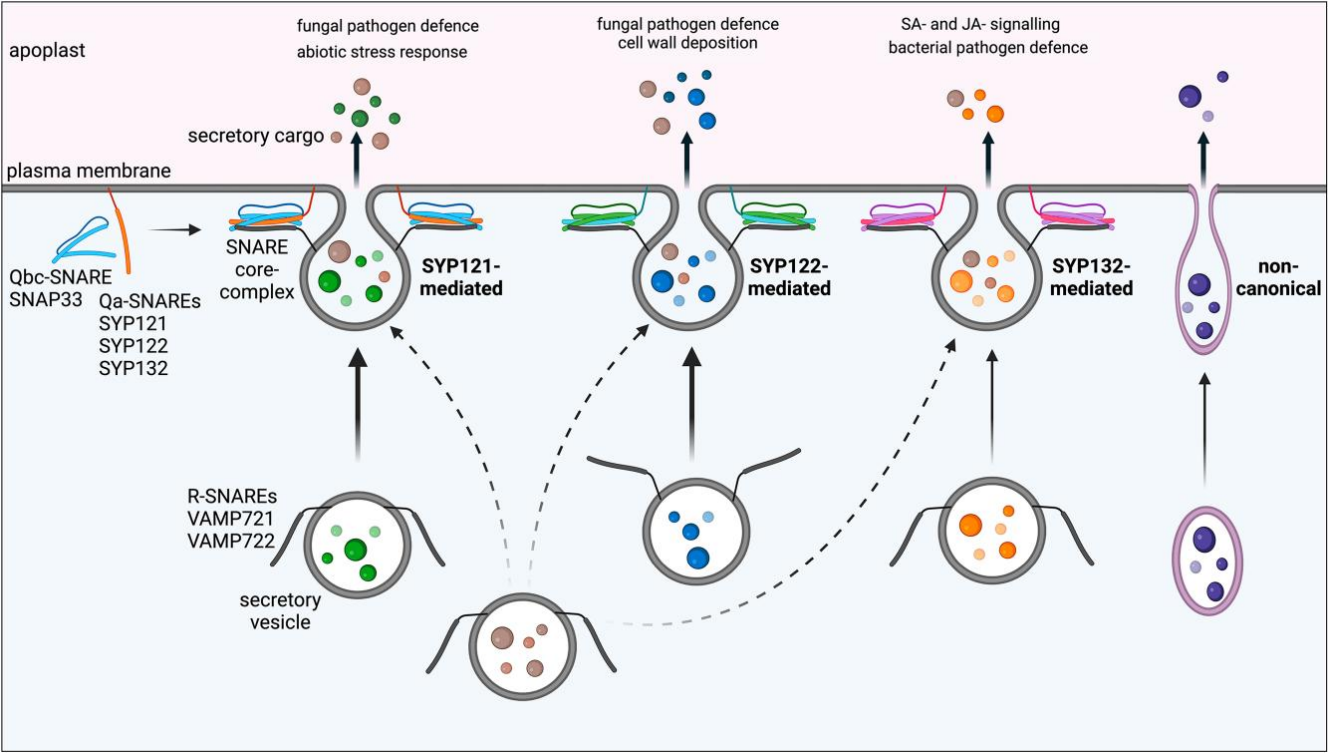
SNARE-assisted secretory trafficking pathways. Schematic summarizing SNARE-assisted secretory vesicle traffic at the PM. Major PM Qa-SNAREs SYP121, SYP122, and SYP132 mediating distinct and overlapping secretory pathways that deliver secretory cargoes to the apoplast are shown. SNARE core complex assemble through binding between PM Qa-SNAREs with Qbc-SNARE (SNAP33) and vesicle-localized R-SNAREs (VAMP721/VAMP722). SYP121- and SYP122-mediated vesicle traffic facilitates a bulk of secretion involved in functions such as fungal pathogen defense, cell wall deposition, and abiotic stress responses. SNARE SYP132 primarily mediates secretion of cargoes involved in bacterial pathogen defense, SA and JA defense signaling mechanisms. A subset of cargoes may be secreted via noncanonical pathways. Created in BioRender. Karnik, R. (2024) BioRender.com/y59p079.

In conclusion (Fig. 4), using advanced quantitative MS, we uncover important roles for Arabidopsis PM SNARE SYP132 in secretory vesicle traffic that governs defense hormone signaling mechanisms impacting basal plant growth and immunity.

## Materials and methods

### Plant lines and propagation

All experiments were performed using fully expanded leaf tissue from *A. thaliana* Columbia-0 ecotype (wild type) and *syp121 (pen1)/syp122* double mutants (Assaad et al. 2004). To manipulate SYP132 function, stably transformed Arabidopsis stable lines were used—35S*CaMV*: RFP-SYP132 (SYP132 overexpressor, SYP132-OX line 1 and line 2) (Xia et al. 2019; Baena et al. 2022). SYP132 overexpression deters or suppresses density and functions of the SNARE at the PM, and this effect is significantly enhanced during pathogen stress (Baena et al. 2022). SYP132-OX plants (above) challenged with pathogens were used in the analysis and are labeled as SYP132^Det^ (=SYP132^Deterrent^; Baena et al. 2024). Plants were grown on soil under standard conditions of 8-h light/16-h dark, 18 °C/22 °C (light/dark) cycle with 150 mmol m^−2^ s^−1^ light and 60% restricted humidity for 3 to 4 wk. The *syp121/syp122* double mutant is highly susceptible to infection and was grown on soil layered with silica (5 mm) to limit contact of the leaves with soil.

### Pathogen infection

Bacterial pathogens *Pst* were cultured as described previously (Tornero and Dangl 2001). Briefly, approximately 24 h before inoculation, the bacteria were plated onto a fresh plate of King's broth agar medium (King et al. 1954) with the appropriate antibiotics and grown for 24 h at 25 °C. Bacteria from plates were suspended in a buffer containing 10 mm MgCl_2_, and OD_600_ of the bacterial suspension was adjusted to 2.5 × 10^5^ cfu mL^−1^. To infect the plants, leaves were poked with a needle and infiltrated abaxially with 10 *μ*L *Pst* inoculum in buffer (10 mm MgCl_2_) or only buffer (control) using a needleless syringe. Pathogen-infected plants were returned to growth chambers with standard growth conditions (above) for 48 h.

### Total RNA isolation and RT-qPCR analysis

Total RNA isolation and RT-qPCR analysis was as described previously (Alexandersson et al. 2005; Xia et al. 2019). Total RNA was extracted using TRIzol Plus RNA Purification Kit (Invitrogen, Paisley, UK). Isolated total RNA was quantified by NanoDrop OneC Microvolume UV-Vis Spectrophotometer (Thermo Fisher Scientific, Leicestershire, UK) before complementary DNA synthesis using QuantiTect Reverse Transcription Kit (Qiagen, Manchester, UK). RT-qPCRs were performed on StepOnePlus Real-Time PCR System (Applied Biosystems, Thermo Fisher Scientific, UK) as per the recommendation of Brilliant III Ultra-Fast SYBR Green qPCR Master Mix (Agilent, Santa Clara, USA) using gene-specific primers (see Supplementary Table S1) (Jang et al. 2004; Armengot et al. 2016). Transcript levels were determined relative to the reference gene, mitochondrial 18S rRNA (AtMg01390). Fold change in the gene expression was calculated as 2^ΔΔCt^ (Livak and Schmittgen 2001) (+/−) *Pst* infection.

### Microsomal membrane purification and fractionation

Isolation of microsomal total membranes and purification of the PM and IM fractions were performed using Minute Plant Plasma Membrane Protein Isolation Kit (Invent Biotech, Plymouth, USA) as per the manufacturer's instruction. The protein quantity of microsomal fractions was determined using Bradford (Bio-Rad Laboratories, Hertfordshire, UK) with BSA Fraction V as a standard. PM and IM pellets were resuspended in 2× Laemmli protein sample buffer (Bio-Rad, UK).

### Apoplast flush extraction

Apoplast flush (secreted proteins) was collected from Arabidopsis leaves as described before (Grefen et al. 2015; Xia et al. 2019; Baena et al. 2022). Briefly, rosette leaves were infiltrated with flush buffer (10 mm MES-KOH, 10 mm MgCl_2_, pH 5.6, protease inhibitor [Thermo Fisher Scientific, UK]) and immediately cut in 1 to 2 strips, about 5 to 6 mm for small leaves, on a cooled platform at 4 °C. The strips were stacked, rolled, and loaded vertically (cut ends top and bottom) in Proteus 1-step Batch Mini Spin Columns (Neo Biotech, CliniSciences, Slough, UK). Apoplast flush was collected using centrifugation at 10,000 × *g* and 4 °C for 1 min. Apoplast flush from 15 to 20 leaves and from 10 to 12 plants was pooled. Total protein in apoplast flush was quantified using Bradford assay (Bio-Rad, UK).

### TMT-MS analysis

The secreted protein pool was trypsin digested, and peptide mixtures were labeled with reagents from TMT-11plex or TMTpro (18plex) TMT isobaric mass tagging kit (Thermo Fisher Scientific, UK), analyzed on electrospray ionization MS. Each sample from the secreted protein pool was digested using the filter-aided sample preparation (FASP) method (Wisniewski et al. 2009) to generate peptides. Peptide mixtures were labeled with reagents from TMT-11plex or TMTpro (18plex) isobaric mass tagging kit (Thermo Fisher Scientific, UK) according to the manufacturer's instructions. Samples were then combined in equal amounts to constitute 11plex or 18plex sets for subsequent MS analysis. LC−MS/MS analysis was performed on a Dionex Ultimate 3000 RSLC nanoflow system (Dionex, Camberley, UK) and Orbitrap Elite mass spectrometer (Thermo Fisher Scientific, UK). TMT-labeled peptide mixtures were loaded on a C18 trap column and then separated on a 50-cm Acclaim PepMap100 column and then separated on a 50-cm Acclaim PepMap100 column (particle size 3 *μ*m, internal diameter 75 *μ*m) with linear gradient 5% to 35% buffer B (0.1% formic acid in 80% acetonitrile) over 135 min at a flow rate of 300 nL/min. Eluate from the column was introduced to the Orbitrap Elite MS by electrospray ionization. The ionization voltage was set to 1.7 kV and the ion transfer tube temperature to 220 °C. The MS was operated in positive ion mode using collision-induced dissociation/higher energy collisional dissociation (CID/HCD) fragmentation methods for MS2. Full scan Fourier transform-based MS (FTMS) spectra were acquired in the range from m/z 380.0 to 1800.0, with a resolution of 60,000. The maximum injection time for the FTMS full scan was set as 200 ms, reaching an automatic gain control (AGC) target value of 1 × 10^6^. The 3 most intense peaks from each MS spectrum were selected for each fragmentation mode. Ions with the charge state 1+ were excluded from the fragmentation list. The HCD MS/MS scan was fixed to start from m/z 100.00 with a resolution of 15,000 using MS2 AGC target of 5 × 10^4^. The collision energy was set as 40% normalized collision energy (NCE). An isolation window of ±1.5 Da was applied to isolate precursor ions with dynamic exclusion of 20 s. Every precursor ion was repeated twice within a duration time of 30 s and was excluded for 20 s. Ion trap MS CID MS/MS scan spectra were acquired with 35% NCE and an AGC target of 1 × 10^4^. Acquired MS/MS spectra were analyzed for protein identification using Proteome Discoverer 3.0 software (Thermo Fisher Scientific, UK).

### MS data analysis

Protein identification and quantification were performed using in silico spectral libraries generated with Proteome Discoverer 3.0 against Arabidopsis taxonomy in NcbiAV (version 2017-1-30; 92,149 sequences). We created 2 spectral libraries for *A. thaliana* proteome, one with modification TMT pro, Carbamidomethyl, Oxidation and a second library with TMT 6plex, Carbamidomethyl, Oxidation using Arabidopsis genome FASTA files, activation type HCD. Spectra were searched against the spectral library with the MSPepSearch node using Proteome Discoverer 3.0. Two missed cleavages for the trypsin digestion were permitted. Identified peptides were filtered with a cutoff criterion of a *q* value of 0.01, corresponding to a 1% false discovery rate (FDR) for highly confident peptide hits and a *q* value of 0.05 (5% FDR) for peptide hits with moderate confidence. For quantitative changes, protein ratios for treatments/control were computed.

Quantification was performed using the abundances of reporter ions based on signal-to-noise ratio values or intensity. Normalization was carried out based on the total peptide amount and scaling on the channel average. For quantitative changes, protein ratios for Arabidopsis lines or treatment vs wild type or control were computed and a protein was determined as differentially abundant within a threshold of log_2_ (fold change) ≥0.5 or ≤−0.5, analyzed by ANOVA.

Protein functional annotation and enrichment analysis including GO analysis of detected proteins describes biological process, and molecular function, and was carried out using PANTHER Overrepresentation Test. All unique genes with biological process information for *A. thaliana* (GO Ontology database DOI: 10.5281/zenodo.7942786, released 2023-05-10) were used as the reference set (all genes in the database).

### Immunoblot analysis

Proteins for immunoblot analysis were suspended in Laemmli buffer and resolved on SDS-PAGE using 4% to 20% gradient gels (Bio-Rad, UK). For immunoblot, proteins resolved on gels were transferred to nitrocellulose membranes (Bio-Rad, UK) using Trans-Blot (Bio-Rad, UK). After incubating in a blocking buffer (1% casein, Bio-Rad, UK), membranes were first probed with primary antibodies: anti-SYP132 (1:3,000 dilution), anti-PR1 (1:10,000 dilution, Agrisera, Vännäs, Sweden), anti-PR2 (1:10,000 dilutions, Agrisera, Sweden), and subsequently, after washing with wash buffer (1× TBS), secondary antibody goat anti-rabbit-horseradish peroxidase conjugate (1:20,000 dilution, Abcam). Primary and secondary antibodies were diluted in 2.5% (w/v) nonfat dry milk, 1× TBS. Proteins were visualized using SuperSignal West Femto Maximum Sensitivity Substrate (Thermo Fisher Scientific, UK) and imaged by Fusion FX Chemiluminescence Imager (Vilber, France). Membranes were stripped in stripping buffer (25 mm glycine-HCI, pH 2.0, 1% SDS) for 60 min with continued agitation and then reprobed to detect additional control proteins. Band density was measured using densitometry using ImageJ (Fiji) software, and values were normalized to the corresponding total protein density for each gel lane detected by Quick Coomassie Stain (Neo Biotech, CliniSciences, UK).

### Statistics

For TMT-MS data, statistical differences were determined using ANOVA. *P* values were calculated by the Fisher test, and their FDRs were adjusted by the Benjamini–Hochberg method. Statistical analysis for data in bar graphs was performed using Prism software (GraphPad). For multiple pairwise comparisons, *P* values are indicated for statistically significant differences using Brown–Forsythe and Welch ANOVA with Dunnett's T3 multiple comparisons or ANOVA with the Holm–Sidak method. Pairwise data analysis was done using 2-tailed Mann–Whitney *t* test (*P* ≤ 0.05).

### Accession numbers

Sequence data from this article can be found in the GenBank/EMBL data libraries under accession numbers SYP132 (At5g08080), SYP121 (At3g11820), and SYP122 (At3g52400). Accession numbers of secreted cargoes can be found in Supplementary Tables S2 to S5).

## Supplementary Material

kiae541_Supplementary_Data

## Data Availability

All relevant data can be found within the manuscript and its supporting materials. The mass spectrometry proteomics data have been deposited to the ProteomeXchange Consortium (http://proteomecentral.proteomexchange.org) via the PRIDE partner repository (Perez-Riverol et al. 2022) with the data set identifier PXD050456.

## Supplementary data

The following materials are available in the online version of this article.

**Supplementary Figure S1.** Analysis of wild type and *syp121/syp122* mutant Arabidopsis.

**Supplementary Figure S2.** Upregulated secretory cargoes and transcript levels.

**Supplementary Figure S3.** SYP132 protein and *PME*, *SYP121*, and *SYP122* transcript levels.

**Supplementary Table S1.** Gene-specific RT-qPCR primer sequences.

**Supplementary Table S2.** List of total proteins detected in wild type and *syp121/syp122* double mutant Arabidopsis secretome.

**Supplementary Table S3.** List of secreted proteins detected in individual SYP132-OX Line1 and SYP132-OX Line2.

**Supplementary Table S4.** List of proteins detected in wild type and SYP132-OX secretory proteome and their classification for association with SA, JA, and auxin signaling pathways.

**Supplementary Table S5.** TMT-MS analysis of secretory proteome from wild type, SYP132-OX, and Arabidopsis.

**Supplementary Table S6.** Secretory cargoes specifically upregulated in *syp121/syp122* double mutant and in SYP132-OX plants.

## Dive Curated Terms

The following phenotypic, genotypic, and functional terms are of significance to the work described in this paper:

VAMP721 Gramene: AT1G04750
VAMP721 Araport: AT1G04750

AHA1 Gramene: AT2G18960
AHA1 Araport: AT2G18960

AIR12 Gramene: AT3G07390
AIR12 Araport: AT3G07390

SNAP33 Gramene: AT5G61210
SNAP33 Araport: AT5G61210

CAT3 Gramene: AT1G20620
CAT3 Araport: AT1G20620

MDH Gramene: AT3G47520
MDH Araport: AT3G47520

SYP122 Gramene: AT3G52400
SYP122 Araport: AT3G52400

PR1 Gramene: TEA004542
PR1 Araport: TEA004542

SYP132 Gramene: At5g08080.1
SYP132 Araport: At5g08080.1

ROS Gramene: reactive oxygen species
ROS Araport: reactive oxygen species

SYP121 Gramene: AT3G11820
SYP121 Araport: AT3G11820

PR2 Gramene: AT3G57260
PR2 Araport: AT3G57260

PME3 Gramene: AT3G14310
PME3 Araport: AT3G14310

PR5 Gramene: AT1G75040
PR5 Araport: AT1G75040

PNP-A Gramene: AT2G18660
PNP-A Araport: AT2G18660

CHI Gramene: AT2G43570
CHI Araport: AT2G43570

CRRSP38 Gramene: AT3G22060
CRRSP38 Araport: AT3G22060

PER34 Gramene: AT3G49120
PER34 Araport: AT3G49120

SBT1.7 Gramene: AT5G67360
SBT1.7 Araport: AT5G67360

LECT6 Gramene: AT5G03350
LECT6 Araport: AT5G03350

Glucose-6-phosphate 1-epimerase Gramene: AT4G25900
Glucose-6-phosphate 1-epimerase Araport: AT4G25900
