# Supplementary material for: SYNTAXIN OF PLANTS 132 underpins secretion of cargoes associated with salicylic acid signaling and pathogen defense
Source: Plant Physiol. 2024 Oct 10;197(1):kiae541. doi: 10.1093/plphys/kiae541 (PMC11663556; doi:10.1093/plphys/kiae541)
Supplement: kiae541_Supplementary_Data [file kiae541_supplementary_data.zip › Supplementary Data.pdf]

Supplemental Figures:

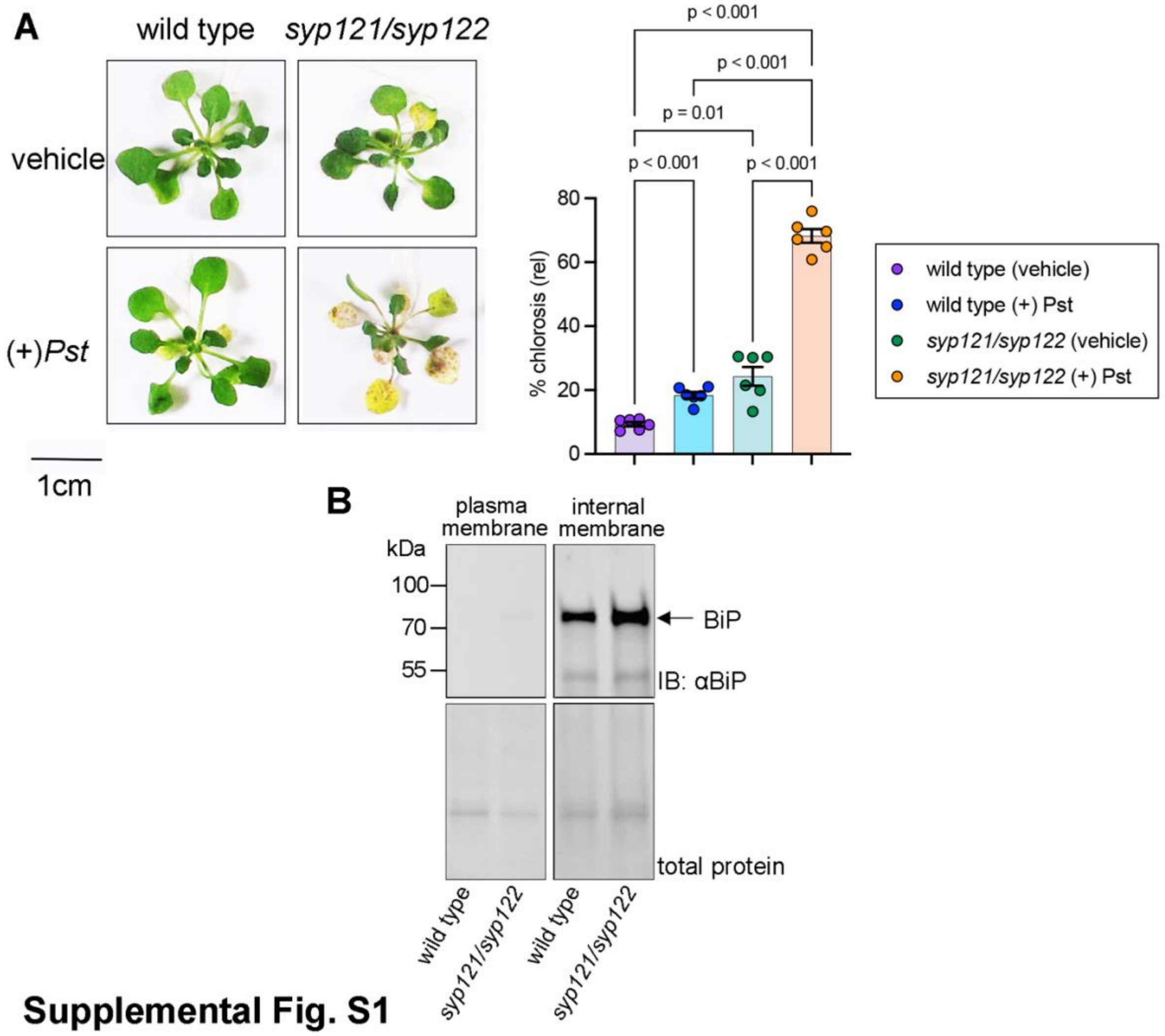

Supplementary Figure S1. Analysis of wild type and *syp121/syp122* mutant Arabidopsis. (c.f Fig.1)

(**A**) Images (representative) of 4-week-old *Arabidopsis thaliana* wild type (left) and *syp121/syp122* mutant (right) following flood inoculation with bacterial pathogens *Pseudomonas syringage* DC3000 [(*Pst*) 2.5 x 10^6 colony-forming units (Cfu) ml^{-1} in 10 mM $MgCl_2$ buffer) or 10 mM $MgCl_2$ buffer (vehicle, control) for five days. Plants were grown of 0.5x Murashige & Skoog media plates with 1% Agar under standard conditions of 8-hour-light/16-hour-dark, 18° C/22° C (light/dark) cycle with 150 mmol $m^{-2}s^{-1}$ photosynthetically active radiation (PAR) light for three weeks prior to treatments. Scale bar =1 cm (for all panels). (c.f Fig.1A). Images were digitally extracted for comparison. Graph (right panel) depicts Mean ±SE (n=6) leaf area with chlorosis relative to area of the whole rosette (measured using Image

J). Statistically significant differences using ANOVA, Dunnett's T3 multiple comparisons test are indicated (p≥0.05 was considered as “not significant”).

(**B**) Immunoblots (representative) of purified plasma membrane and internal membrane fractions from wild type and *syp121/syp122* mutant Arabidopsis leaf tissue. BiP is an internal membrane resident protein. Proteins were resolved using SDS-PAGE and Lumen-binding protein BiP (~ 74 kDa) was detected using anti-BiP antibodies. Purity of the plasma membrane fractions was estimated at >99% using the BiP band density in internal membranes as a reference. Coomassie staining of immunoblot membranes (bottom panels) shows total protein. Black lines (left) indicate positions of molecular mass markers, and black arrows (right) mark expected band positions.

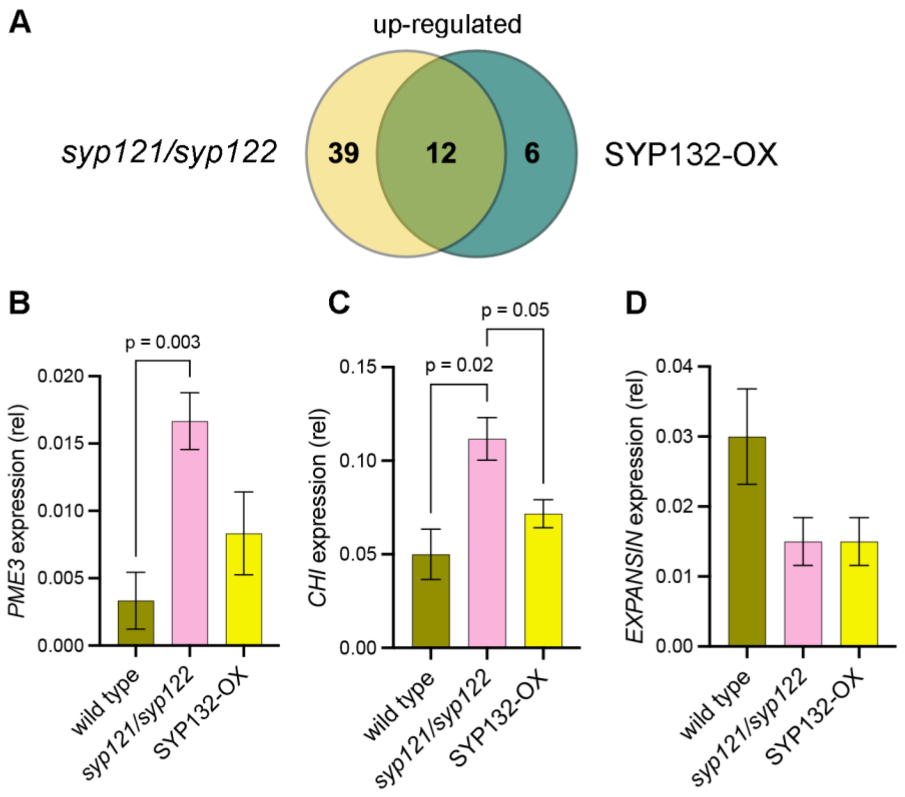

Supplemental Fig. S2

Supplementary Figure S2. Up-regulated secretory cargoes and transcript levels. (c.f Fig.2)

(**A**) Venn diagram depicting the number of significantly upregulated secreted cargoes relative to the wild type plants in *syp121/syp122* double mutant (Fig.1H) that specifically overlap with high

abundant cargoes detected in SYP132-OX plants (Fig.2A). (**B-D**) Mean ±SE (n=3) gene expression in wild type, *syp121/syp122* mutant and SYP132-OX Arabidopsis leaf tissue relative to 18S using RT-qPCR with gene specific primers (c.f Supplemental Table 1) detecting defence-related *PME3* (**B**), *ENDOCHITINASE* (**C**), and as control, *EXPANSIN-LIKE A1* (**D**). Data are mean ± SE (n=3). Statistically significant differences using Brown-Forsythe and Welch ANOVA with Dunnett's T3 multiple comparisons are indicated ($p \geq 0.05$ was considered as "not significant").

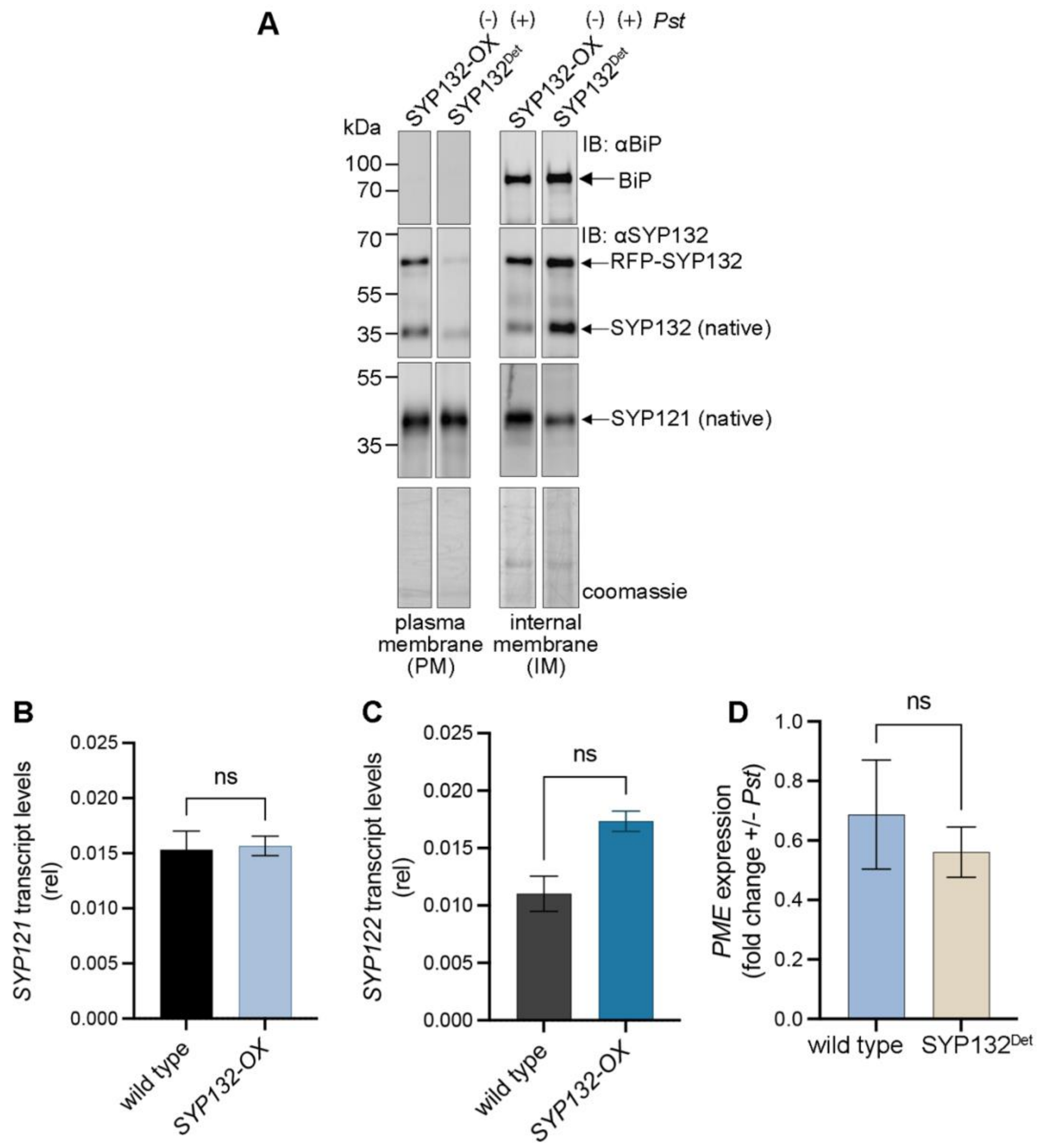

Supplemental Fig. S3

Supplementary Figure S3. SYP132 protein and *PME*, *SYP121*, *SYP122* transcript levels. (c.f Fig.3)

(**A**) Immunoblots (representative) of purified plasma membrane (left panels) and internal membrane proteins (right panels) derived from leaf tissue of SYP132-OX [control, 35S:RFP-SYP132] compared against SYP132^{Deterrent} [SYP132^{DT}, (+)*Pst*]. Proteins were resolved using SDS-PAGE. Proteins were resolved using SDS-PAGE. Total SYP132 protein native (~35 kDa) + RFP-SYP132 (~61 kDa) were detected using anti-SYP132 antibodies. Native SYP121 protein (~38 kDa) was detected using anti-SYP121 antibodies. Lumen-binding protein BiP (~74 kDa), a marker for internal membranes using anti-BiP antibodies. Purity of the plasma membrane fractions using BiP bands as reference was estimated at >99%. Coomassie stained membranes detect total protein. Black lines (left) indicate positions of molecular mass markers, and black arrows (right) indicate expected band positions.

(**B-C**) Mean ± SE (n=3) gene expression in wild type and SYP132-OX Arabidopsis leaf tissue relative to 18S using RT-qPCR with gene specific primers (c.f Supplemental Table 1) detecting *SYP121* (**B**) and *SYP122* (**C**) transcripts. Data are mean ± SE (n=3). Statistical significance using two-tailed Mann Whitney t-test is indicated ($p \geq 0.05$ was considered as "not significant").

(**D**) Fold change in *PME3* expression in Arabidopsis wild type and SYP132^{Det} leaf tissue following infection with bacterial pathogens *Pseudomonas syringae* relative to buffer (10 mM $MgCl_2$) treatment (+/- *Pst*). Data are $2^{\Delta\Delta Ct}$ relative to 18S using RT-qPCR with gene specific primers (Supplemental Table 1). Statistical significance using Brown-Forsythe and Welch ANOVA as indicated ($p \geq 0.05$ was considered as "not significant"). Expression of an additional defence-related gene *PME3* was determined (c.f Fig.3B).
